# Emerging Implications of Phase Separation in Cancer

**DOI:** 10.1002/advs.202202855

**Published:** 2022-09-18

**Authors:** Jiang Ren, Zhenyu Zhang, Zhi Zong, Long Zhang, Fangfang Zhou

**Affiliations:** ^1^ School of Medicine Zhejiang University City College Hangzhou 215123 China; ^2^ The Eighth Affiliated Hospital Sun Yat‐sen University Shenzhen 518033 China; ^3^ Department of Neurosurgery The First Affiliated Hospital of Zhengzhou University Zhengzhou Henan 450003 China; ^4^ MOE Key Laboratory of Biosystems Homeostasis & Protection and Innovation Center for Cell Signaling Network Life Sciences Institute Zhejiang University Hangzhou 310058 China; ^5^ International Biomed‐X Research Center, Second Affiliated Hospital of Zhejiang University School of Medicine Zhejiang University Hangzhou China; ^6^ Cancer Center Zhejiang University Hangzhou 215123 China; ^7^ Institutes of Biology and Medical Sciences Soochow University Suzhou 215123 China

**Keywords:** biomolecular condensates, cancer, novel therapeutics, phase separation

## Abstract

In eukaryotic cells, biological activities are executed in distinct cellular compartments or organelles. Canonical organelles with membrane‐bound structures are well understood. Cells also inherently contain versatile membrane‐less organelles (MLOs) that feature liquid or gel‐like bodies. A biophysical process termed liquid–liquid phase separation (LLPS) elucidates how MLOs form through dynamic biomolecule assembly. LLPS‐related molecules often have multivalency, which is essential for low‐affinity inter‐ or intra‐molecule interactions to trigger phase separation. Accumulating evidence shows that LLPS concentrates and organizes desired molecules or segregates unneeded molecules in cells. Thus, MLOs have tunable functional specificity in response to environmental stimuli and metabolic processes. Aberrant LLPS is widely associated with several hallmarks of cancer, including sustained proliferative signaling, growth suppressor evasion, cell death resistance, telomere maintenance, DNA damage repair, etc. Insights into the molecular mechanisms of LLPS provide new insights into cancer therapeutics. Here, the current understanding of the emerging concepts of LLPS and its involvement in cancer are comprehensively reviewed.

## Graphical abstract

Liquid–liquid phase separation (LLPS) elucidates how membrane‐less organelles (MLOs) form through dynamic biomolecules assembly. In this review, the concept of LLPS and how it promotes MLOs formation are described. Then, the current understanding of LLPS regulation is presented. How LLPS/MLOs are involved in cancer are also discussed. Finally, potential methods are described to interfere with aberrant LLPS/MLOs.

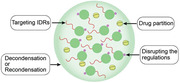

## 1 Introduction

The world inside cells is hectic as they must manage numerous molecular events in an ordered manner. Eukaryotic cells evolutionarily developed optimal inner structures, compartments, or organelles with distinct properties and functions to orchestrate biochemical reactions while allowing spatiotemporal and hierarchical control.^[^ ^1^, ^2^ ^]^ In canonical membrane‐bound organelles (MBOs) such as the nucleus, mitochondria, endoplasmic reticulum, Golgi apparatus, lysosomes, and secretory vesicles, the exterior and interior environments are physically separated by an enclosed lipid bilayer membrane. MBO components are transported and localized using specialized passive and active trafficking machinery.^[^ ^1^, ^2^ ^]^

**Figure 1:**
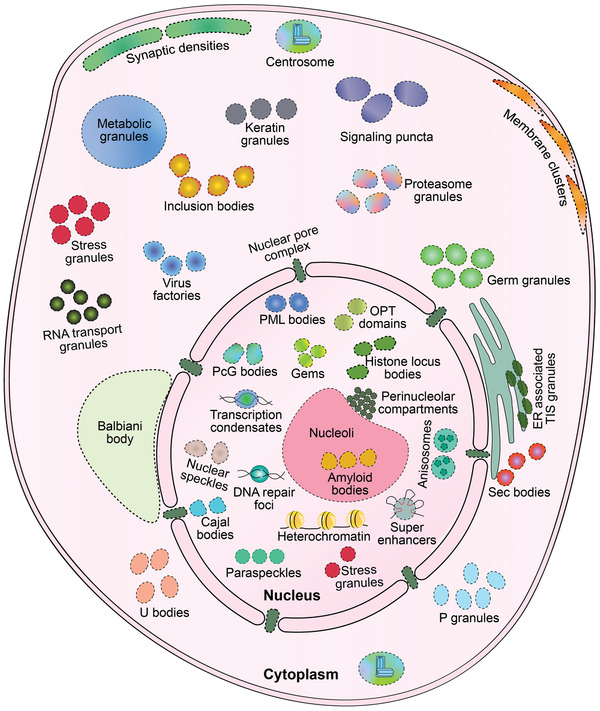
Schematic diagram showing biomolecular condensates localization in a eukaryotic cell. Dashed lines indicate the border of membraneless organelles (MLOs). Note that some condensates only exist in specific cells. See the following references for detailed information regarding individual condensates. Cytoplasm: Balbiani body in oocytes,^[^ ^5^ ^]^ centrosomes,^[^ ^7^ ^]^ ER‐associated TIS granules,^[^ ^283^ ^]^ germ granules in germ cells,^[^ ^8^ ^]^ inclusion bodies in neurons,^[^ ^284^ ^]^ keratin granules in keratinocytes,^[^ ^285^ ^]^ membrane clusters,^[^ ^286^, ^287^ ^]^ metabolic granules,^[^ ^194^ ^]^ P granules,^[^ ^13^ ^]^ proteasome granules,^[^ ^288^ ^]^ RNA transport granules in neurons,^[^ ^289^ ^]^ sec bodies,^[^ ^290^ ^]^ signaling puncta,^[^ ^55^, ^183^, ^184^ ^]^ stress granules,^[^ ^15^, ^16^ ^]^ synaptic densities in neurons,^[^ ^291^ ^]^ U bodies,^[^ ^292^ ^]^ virus factories in virus‐infected cells;^[^ ^293^ ^]^ Nucleus: anisosomes,^[^ ^34^ ^]^ amyloid bodies,^[^ ^202^ ^]^ Cajal bodies,^[^ ^6^ ^]^ DNA repair foci,^[^ ^227^ ^]^ Gemini of Cajal bodies (Gems),^[^ ^294^ ^]^ heterochromatin,^[^ ^9^ ^]^ histone locus bodies,^[^ ^6^ ^]^ nuclear speckles,^[^ ^11^ ^]^ nucleoli,^[^ ^10^ ^]^ oligopeptides transporter (OPT) domains,^[^ ^295^ ^]^ paraspeckles,^[^ ^12^ ^]^ Polycomb group (PcG) bodies,^[^ ^296^ ^]^ perinucleolar compartments,^[^ ^297^ ^]^ promyelocytic leukemia (PML) bodies,^[^ ^14^ ^]^ stress granules,^[^ ^15^, ^16^ ^]^ super enhancers,^[^ ^17^ ^]^ transcription condensates;^[^ ^233^, ^234^, ^235^ ^]^ and the nuclear pore complex embedded in the nuclear membrane.^[^ ^5^ ^]^

Despite MBOs being described as paradigmatic organelles, some membraneless organelles (MLOs) were observed over a century ago. Among these, the nucleolus is the most well‐known organelle.^[^ ^3^, ^4^ ^]^ With advanced experimental methodologies and techniques, studies on MLOs began to boom approximately a decade ago, which has increased our understanding of MLOs with distinct constituents and functions. As shown in **Figure** **1**, MLOs include Balbiani body,^[^ ^5^ ^]^ Cajal bodies,^[^ ^6^ ^]^ centrosomes,^[^ ^7^ ^]^ germ granules,^[^ ^8^ ^]^ heterochromatin,^[^ ^9^ ^]^ nucleoli,^[^ ^10^ ^]^ nuclear speckles,^[^ ^11^ ^]^ paraspeckles,^[^ ^12^ ^]^ P granules,^[^ ^13^ ^]^ promyelocytic leukemia (PML) bodies,^[^ ^14^ ^]^ stress granules (SGs),^[^ ^15^, ^16^ ^]^ superenhancers,^[^ ^17^ ^]^ etc. MLOs are located in the nucleus or cytoplasm, and most are rich in proteins and/or nucleic acids. MLOs are also referred to as biomolecular condensates whose diameters range from 0.1 to 3 µm.^[^ ^18^ ^]^ MLOs perform diverse functions in cellular organization, signaling, puncta formation and transduction, ribosome biogenesis, mitosis, and asymmetric cell division.^[^ ^19^ ^]^

MLOs discovery depends on spontaneous assembly via a biophysical process termed liquid‐liquid phase separation (LLPS). The cellular environment is usually considered to be aqueous. In this scenario, functional MLOs components are selectively partitioned and highly concentrated in liquid‐like droplets.^[^ ^18^ ^]^ The MLOs components are dynamically exchanged with the surrounding aqueous milieu in a mixing/demixing manner. Thus, specific biochemical reactions can be facilitated or inhibited. It is worth noting that solid‐like hydrogels can be separated from bulk solvent, except for LLPS droplets. Therefore, MLOs can also behave as dynamic hydrogel‐ or non‐dynamic solid‐like structures.^[^ ^20^ ^]^

Previous studies of LLPS and MLOs have revealed insights into the molecular pathogenesis of age‐related diseases. Typically, aberrant protein aggregation caused by LLPS dysfunction is increasingly demonstrated as a key problem in cancer. In this review, we describe the concept of LLPS and how it promotes MLOs formation. Then, we present the current understanding of LLPS regulation. We also discuss how LLPS/MLOs are involved in cancer. Finally, we describe potential methods to interfere with aberrant LLPS/MLOs.

## 2 The Concept of LLPS and Phase Transition in Biological Systems

**Figure 2:**
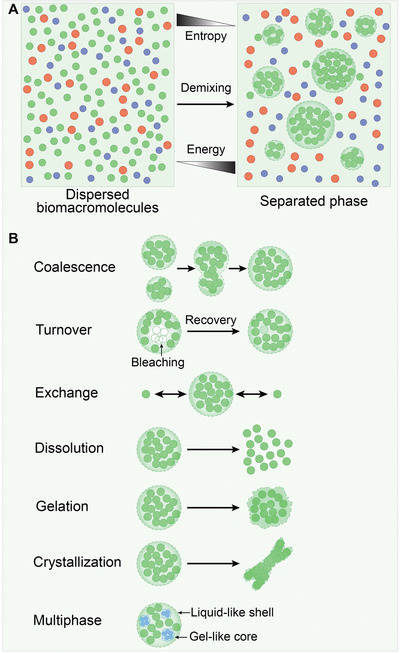
Representation and characteristics of liquid–liquid phase separation (LLPS). A) Entropy typically drives biomacromolecules dispersion in a heterogeneous solution. A subset of multivalent molecules can overcome entropic effects and spontaneously condense into distinct liquid‐like (droplet) phases at the cost of energy. B) Phase‐separated droplets coalescence into larger droplets. Molecules within the liquid droplet are high turnover that can be examined by fluorescence recovery after photobleaching (FRAP) experiments. Meanwhile, droplet components are rapidly exchanged with surrounding solution. Certain changes in the solution conditions can lead to droplet dissolution. In addition, liquid‐like phases can progressively transition into less fluid gel‐ and fibril‐like solid states with less fluidity. Multiphase droplets contain a reversible liquid‐like shell outside and several immiscible gel‐like cores inside.

LLPS is a widespread phenomenon in nature. In LLPS, two or more immiscible components in a solvent tend to spontaneously and rapidly demix into distinct phases that can stably coexist.^[^ ^21^, ^22^ ^]^ For example, after shaking a mixture of oil and water, the oil disperses from the surrounding water to form droplets that quickly float and coalesce with each other upon contact. The mechanical surface tension at the boundary between the two aqueous phases governs and shapes the spherical droplets.^[^ ^22^ ^]^ Generally, LLPS is reversible, making the process tunable. Therefore, biological LLPS occurs when proteins alone or together with nucleic acids and other biomacromolecules (lipids and carbohydrates) from the surrounding aqueous‐like cellular environment form a distinct liquid‐like phase (**Figure** **2**A). Various strategies have been applied to experimental analysis of LLPS behavior. Generally, in vitro LLPS is observed by droplets formation using a fully defined set of components (pure proteins or/and nucleic acids). Meanwhile, in‐cell biomolecular condensates can be reconstituted by directly expressing fluorescently tagged proteins or gene editing‐mediated tagging of endogenous proteins. Proximity‐labeling techniques enable the profiling of condensate components.^[^ ^23^, ^24^ ^]^ The physical characteristics can be investigated via microscopic imaging. A standard example is observation of fusion/coalescence events to demonstrate the liquid‐like behavior of dense phase using differential interference contrast or fluorescence microscopy. Fluorescence recovery after photobleaching (FRAP) experiments and fluorescence correlation spectroscopy are typically used to measure the dynamics diffusion/turnover within condensates and molecular exchange with the surrounding bulk phase.^[^ ^23^, ^24^ ^]^

Consistent with descriptions of LLPS, a seminal study demonstrated that P granules are MLOs that are shaped by biological LLPS and exhibit typical liquid‐like droplets in *Caenorhabditis elegans* embryos.^[^ ^13^ ^]^ FRAP experiments showed that proteins within P granules rearrange quickly and exchange components rapidly with the surrounding cytoplasm.^[^ ^13^ ^]^ Another FRAP examination of metabolically active nucleoli in *Xenopus laevis* oocytes showed that these nucleoli are liquid‐like MLOs with rapid coalescence and turnover.^[^ ^25^ ^]^ Based on the principles of quick rearrangement, coalescence, turnover, exchange and dissolution (Figure 2B), an increasing number of liquid‐like MLOs were identified in subsequent years.

Broadly, matters can exist as a liquid, solid, gas, or plasma (under certain conditions). When conditions change, matter can undergo a phase transition from one state to another. A common example is the phase transition of water, which can exist as liquid, ice (condensed solid), or vapor (dilute gas). Here in cell biology, we highlight liquid‐to‐solid transitions such as gelation or crystallization (Figure 2B). Actually, LLPS can be considered as a special type of phase transition, that is, a density transition that results in condensed phase formation.^[^ ^26^ ^]^ Furthermore, LLPS, gelation, and crystallization are frequently linked. One vital argument for this association is that high‐density proteins (i.e., a condensed phase) has a strong thermodynamic tendency to transition into a viscous liquid or even a hydrogel/crystal‐like phase.^[^ ^26^, ^27^, ^28^ ^]^ This kind of transition is often utilized by structural biologists for protein crystallization. Gel‐like MLOs, such as the Balbiani body, nuclear pore complexes, and pericentriolar material, are less dynamic during inter‐coalescence, intra‐rearrangement, and components exchange.^[^ ^5^ ^]^ Even more complicated, intensive studies on the structure of SGs, nuclear bodies, Cajal bodies, nuclear speckles, nucleoli, and anisosomes show that these structures contain a reversible liquid‐like outer shell and several inner immiscible gel‐like sub‐compartments/cores called multiphases (Figure 2B).^[^ ^10^, ^29^, ^30^, ^31^, ^32^, ^33^, ^34^ ^]^ The shell and cores have distinct compositions, viscosities, and surface tensions that may reflect the reason underlying multiphase coexistence. A phase with lower surface tension can encapsulate a phase with higher surface tension.^[^ ^10^, ^35^, ^36^ ^]^ However, it remains elusive how the formation of such multiphase MLOs is primed and regulated. Additionally, the function of the sub‐compartments remains unclear as well.

## 3 The Force Driving Phase Separation Is Likely Embedded in Protein and Nucleic Acid Sequences

According to the second law of thermodynamics, the entropy increases in any spontaneous process. Thus, demixing is an anti‐entropy process that decreases the solubility of miscible molecules at the cost of energy consuming (Figure 2A). This observation implies that biomacromolecules tend towards a state of disorder distribution in cells. Whereas, in the theories of polymer science, mixed multivalent molecules can overcome the entropic effect and spontaneously assemble into larger oligomers or polymers through numerous multivalently mediated weak interactions.^[^ ^18^, ^19^, ^22^ ^]^ Previous studies showed that ATP can serve as an energy source for these interactions.^[^ ^29^ ^]^ These two theoretical bases are widely used to describe the mechanisms of biological LLPS and MLOs formation. In combination with the well‐accepted driver (or scaffold)/client concept, the molecules driving LLPS include proteins, DNA, and RNA. These molecules often have multi‐valency, which mediates low‐affinity inter‐ or intra‐molecule interactions that trigger phase separation and attract client molecules for condensate partitioning. LLPS cannot occur or formed MLOs are unstable if driver molecules are depleted.^[^ ^14^, ^37^, ^38^, ^39^, ^40^ ^]^ What's impressive, multi‐valency‐mediated weak protein organization is designated as quinary structures inside the cells that define a fifth level of protein complexity beyond quaternary structures.^[^ ^41^, ^42^ ^]^

**Figure 3:**
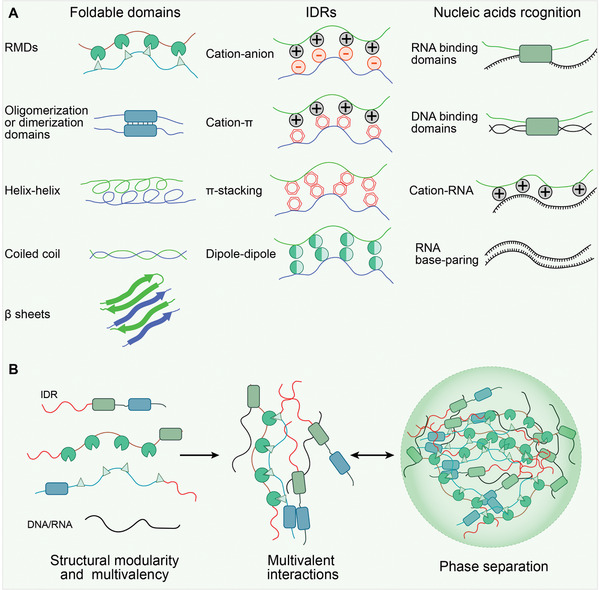
The underlying mechanisms that drive protein phase separation. A) Several molecular features have been identified in phase separation proteins. These features mediate weak transient interactions between multivalent proteins, including foldable domain interactions (repetitive modular domains (RMDs), oligomerization or dimerization domains, helix‐helix, coiled coil, *β*‐sheets), intrinsically disordered regions (IDRs) interactions (cation–anion, cation–*π*, *π*–stacking, dipole–dipole), and nucleic acids recognition domains interactions (DNA/RNA binding domains, cation‐RNA, RNA base‐paring). B) Overview of multivalent interactions‐driven condensate assembly.

Several structural features including weakly adhesive multivalent motifs, repetitive modular domains (RMDs), oligomerization or dimerization domains, intrinsically‐disordered regions (IDRs), and nucleic acid recognition domains have been identified in driver proteins undergoing LLPS.^[^ ^14^, ^37^, ^38^, ^43^, ^44^ ^]^ Driver proteins also typically possess multicombination motifs. Cooperatively, a diverse range of forces participate in motif interactions and drive phase separation, such as electrostatic interactions (cation–anion), *π*‐effects (cation–*π*, *π*‑stacking), Van der Waals forces (dipole–dipole), and *β* sheets (**Figure** **3**).^[^ ^27^, ^38^, ^39^, ^45^, ^46^, ^47^, ^48^, ^49^, ^50^, ^51^ ^]^ Worth to note, a comprehensive work presented an integrative database named DrLLPS which provides rich annotations on the properties of LLPS‐associated proteins in 164 eukaryotic species by compiling and integrating the knowledge from widely used public resources.^[^ ^52^ ^]^

### 3.1 Foldable Domains

RMDs in proteins that drive MLOs formation are connected by flexible linkers. These RMDs mediate stereospecific and electrostatic interactions between proteins, thereby forming a complex network. as exemplified in the multivalent Src homology 3 (polySH3)/proline‐rich motif (polyPRM) system, which is linked by SH3‐domain repeats that bind PRMs.^[^ ^37^, ^53^, ^54^ ^]^ Observations using artificial proteins showed that liquid‐like droplets formed once the tandem polySH3‐containing protein constructs were mixed with complementary polyPRM‐containing protein constructs. Expressing these two constructs in living cells also results in LLPS and liquid‐like droplet formation.^[^ ^37^ ^]^ There is also evidence that LLPS is guided by endogenous ploySH3/polyPRM systems. In the case of actin polymerization, SH3 in the noncatalytic region of tyrosine kinase (NCK) interacts with a PRM in neural Wiskott‐Aldrich syndrome protein (N‑WASP). Additionally, SH2 in NCK interacts with phosphotyrosine residues in the adhesion receptor Nephrin. Through multivalent interactions, the Nephrin/NCK/N‐WASP multivalent protein system can assemble and phase‐separate into liquid‐like droplets in solution^[^ ^37^ ^]^ or clusters on lipid bilayers.^[^ ^54^ ^]^ Another polySH3/polyPRM system is implied by the phase‐separated microcluster formation of linker for activation of T cell (LAT) proteins on plasma membranes, which occurs upstream of Nephrin/NCK/N‐WASP interaction events. In this system, multivalent tyrosine‐phosphorylated LAT, SH2, SH3‐containing growth factor receptor‐bound protein 2 (GRB2), and PRM‐containing son of sevenless 1 (SOS1) function as a dock, an adaptor, and a binding partner, respectively.^[^ ^55^ ^]^ Similar to the SH3/PRM system, another complex involves repetitive domains of small ubiquitin‐like modifier (polySUMO) and the polySUMO interacting motif (polySIM). The polySUMO/polySIM system is presented in P‐bodies and nuclear PML bodies.^[^ ^14^, ^56^ ^]^ These studies finally conclude that the higher valence behind more modular domains result in a higher possibility of phase separation and complex assembly.

In addition, folded, nonrepetitive oligomerization or dimerization domains are found in proteins that are also crucial for MLOs formation through self‐interaction. Some examples include RAS GTPase‐activating protein‐binding protein 1 (G3BP1), transactive response DNA binding protein 43 kDa (TDP43) in SGs, the PML protein in PML bodies, coilin in Cajal bodies, and speckle‐type pox virus and zinc finger protein (SPOP) in nuclear speckles.^[^ ^57^, ^58^, ^59^, ^60^, ^61^ ^]^ The sterile alpha motif (SAM) domain of the polycomb protein SOP2 mediates oligomerization, thereby accelerating SOP2 condensate gelation.^[^ ^62^ ^]^ An “optoDroplet” system was created by fusing the light‐sensitive oligomerization domain (the photolyase homology region) of *Arabidopsis thaliana* cryptochrome 2 with IDRs of various proteins. Using this system, light‐activated artificial protein oligomerization drives rapid assembly of intracellular liquid droplet‐like clusters.^[^ ^63^ ^]^

### 3.2 Low Complexity Domains (LCDs)

Approximately 33–55% of eukaryotic proteomes are predicted to have IDRs.^[^ ^64^, ^65^ ^]^ Compared to structured proteins, IDRs have an intrinsically versatile and flexible conformation because they lack hydrophobic amino acids, which are required for highly ordered folding in stable secondary and tertiary structures.^[^ ^65^ ^]^ Although some IDRs can adopt stable tertiary structures upon interacting with other macromolecules, there is no evidence showing folding‐upon‐interacting of IDRs when analyzing proteins within liquid‐like droplets using nuclear magnetic resonance (NMR).^[^ ^39^, ^66^ ^]^ The MLO‐associated IDRs have a few LCDs which can be identified by some online tools such as MobiDB, DisMeta, PONDR, PLAAC, IUPred3, etc., with predictive algorithms according to amino acid composition, frequency, and distribution.^[^ ^24^, ^37^ ^]^

One subset of LCDs, prion‐like domains (PrLDs), are dominated by hydrophilic amino acids with uncharged polar side chains (glutamine, glycine, asparagine, tyrosine, serine) for assembling amyloid‐like aggregates in a self‐templating manner.^[^ ^67^ ^]^ Many PrLD‐containing proteins were early identified in yeast; MOT3, RNQ1, SWI1, SUP35, and URE2 are the best characterized in prion formations when they are expressed at high levels.^[^ ^68^, ^69^, ^70^ ^]^ In addition, the Balbiani bodies present in early stage *Xenopus laevis* oocytes via amyloid‐like self‐assembly of the *Xenopus* homolog of Bucky ball (XVelo) proteins, which is dictated by N‐terminal PrLDs.^[^ ^47^ ^]^ In the human genome, 240 protein‐encoding proteins that harbor a domain strongly analogous to annotate yeast PrLDs were identified using predictive algorithms.^[^ ^70^, ^71^ ^]^ Of these, 72 are labeled as RNA‐binding proteins (RBPs), including amyotrophic lateral sclerosis (ALS), Ataxin1/2, Ewing sarcoma breakpoint region 1 (WSR1), TDP43, human heterogeneous nuclear ribonucleoprotein A 1/2 (hnRNPA1/2), and the TET/FET protein family (contains three members: translocated in liposarcoma (TLS)/fused in sarcoma (FUS), Ewing's sarcoma oncoprotein (EWS) and TATA‐box binding protein associated factor 15 (TAF15)), etc. These proteins are aggregation‐prone and act as driver proteins for hydrogel‐like ribonucleoprotein (RNP) granule formation by working together with RNA species.^[^ ^28^, ^70^, ^72^, ^73^, ^74^, ^75^ ^]^ High‐resolution electron microscopy studies revealed that such hydrogels are composed of protein fibrils.^[^ ^76^, ^77^, ^78^ ^]^ Protein fibrils can be extended to protofilaments via weak cross‐*β* sheet interactions through polar and aromatic side chains, thus conferring a less stable hydrogel‐like RNP granule structure under physiological conditions, i.e., lability.^[^ ^38^, ^76^, ^77^, ^78^, ^79^ ^]^ Among the reasons, multiple short structured motifs within LCDs called low‐complexity, aromatic‐rich, kinked segments (LARKS) provide weak Velcro‐like adhesions between LCDs, as seen in the N‐terminal of FUS.^[^ ^76^, ^78^ ^]^ Additionally, multiple short linear motifs (SLiMs) found in the LCDs of TDP43, hnRNPA1, and decapping mRNA 2 (Dcp2) etc. can mediate the formation of stronger steric *β*‐zippers and less labile interactions with well‐folded domains of other proteins.^[^ ^80^, ^81^, ^82^, ^83^ ^]^

Unlike PrLD‐associated LCDs, another subset of LCDs contains disproportionately high negative and/or positive amino acids (mostly arginine, lysine, glutamic acid, and aspartic acid), particularly short arginine/glycine (RGG or GRG) repeats, which generate negative/positive‐charged blocks.^[^ ^39^, ^45^, ^46^ ^]^ The term “complex coacervation” is used here to describe electrostatic interactions and condensation between multiple pairs of highly and oppositely charged proteins or proteins (positive) and nucleic acids (negative). These interactions are particularly important for RNP granules, chromatin, and nucleoli formation.^[^ ^48^, ^84^, ^85^, ^86^ ^]^

Interestingly, the protein sequences in MLOs are often interspersed with tyrosine and phenylalanine aromatic residues between LCDs that mediate short‐range *π*‐stacking interactions and cation–*π* interactions with arginine or lysine residues, thereby promoting phase separation.^[^ ^38^, ^46^, ^48^, ^87^ ^]^ For example, FUS contains polar and aromatic residues that form cation–*π* (arginine‐tyrosine) interactions and facilitate phase separation.^[^ ^88^, ^89^ ^]^ The aromatic rings of the phenylalanine‐glycine (FG) repeats in DEAD box helicase 4 (DDX4) are engaged in both cation–*π* and *π*–stacking interactions with tyrosine residues and promote phase separation.^[^ ^51^, ^90^ ^]^ In addition, hydrophobic and dipolar‐dipolar interactions independent of the LCD amino acid composition were also proposed as driving forces for phase separation.^[^ ^87^, ^91^, ^92^, ^93^ ^]^

### 3.3 Recognition of Nucleic Acids

In addition to proteins, nucleic acids including nascent RNA, or long noncoding RNA (lncRNA), or genome DNA with repetitive sequences are enriched in most MLOs and are required for their formation.^[^ ^94^ ^]^ Nucleic acids are highly negatively charged because of their phosphate‐based anionic backbones. Thus, electrostatic interactions are the main force involved in the phase separation of nucleic acids. In addition, different RNA moieties are recognized by some RNA‐binding modular domains in RBPs, such as 1) RNA recognition motifs (RRMs) in WHI3, T‐cell intercellular antigen 1 (TIA1), polypyrimidine tract‐binding (PTB), and the N‐terminal of hnRNPA1/2 proteins;^[^ ^27^, ^37^, ^95^, ^96^, ^97^ ^]^ 2) zinc fingers (ZnFs) in EWS, TAF15, and FUS proteins;^[^ ^98^, ^99^ ^]^ 3) polyglutamine (polyQ) tracts in WHI3 protein;^[^ ^96^, ^97^ ^]^ and 4) double‐stranded RNA‐binding domains (dsRBDs). Correspondingly, RNAs have repetitive poly‐CUG, CAG, CGG, CCUG, and GGGGCC sequences in coding/noncoding regions transcribed from repetitive DNA sequences.^[^ ^100^, ^101^ ^]^ Repetitive RNAs work as docks to recruit and sequester specific RBPs by adopting versatile secondary structures such as (semi‐)stable hairpins or more stable G‐quadruplexes formed through (non‐)canonical Watson‐Crick base‐pairing and helical stacking.^[^ ^97^, ^100^, ^102^, ^103^, ^104^ ^]^ Moreover, intermolecular RNA‐RNA interactions can mediate protein‐free RNA self‐assembly and promote phase separation.^[^ ^100^ ^]^ Previous studies showed that RNA length, secondary structure, and RBP:RNA stoichiometry are major determinants of RNP granule formation and identity. The contributions of protein‐protein, protein‐RNA, and RNA‐RNA interactions to RNP granule formation are relative and vary among different RNP granules. Thus, RNP granule formation can be classified as protein‐driven, RNA‐driven, and combination. These processes are reviewed elsewhere.^[^ ^94^, ^104^ ^]^

Some DNA‐containing MLOs have been identified, including constitutive heterochromatin, polycomb group (PcG) bodies (facultative heterochromatin), chromatin, nucleosomes, nucleoli, telomeres, transposons, CCCTC‐binding factor (CTCF) clusters, superenhancers, transcription factories, DNA replication origins, Barr bodies, and DNA damage foci. These MLOs occur at specific genomic loci where a large number of DNA motifs/elements contribute to locus‐specific and high‐affinity protein‐DNA interactions.^[^ ^105^, ^106^, ^107^, ^108^ ^]^ Most transcription factors (TFs) are bifunctional proteins that contain at least one folded domain for DNA sequence‐specific binding and an LCD for weak interactions between other LCD cofactors.^[^ ^109^ ^]^ One model of condensates/factories/hubs proposes that TFs bind to cis‐regulatory elements such as promoters, enhancers, and other components of the transcription machinery, thereby leading to phase separation and the assembly of liquid transcription compartments.^[^ ^109^, ^110^, ^111^ ^]^ In cytoplasm, exogenous DNA binding to cyclic GMP‐AMP synthase (cGAS) in the cytoplasm robustly induces the formation of liquid‐like droplets, in which cGAS is activated.^[^ ^112^ ^]^

A large portion of repetitive DNA sequences is deployed at stretches of DNA between genes in eukaryotic genomes. In contrast to the previous hypothesis that these sequences comprise junk DNA, repetitive DNA sequences are essential for genome function and are packaged into a dense heterochromatin structure. The mechanism of heterochromatin formation has been implicated in phase separation as well.^[^ ^113^ ^]^

## 4 Regulation of Phase Separation

Responsiveness to environmental stimuli and metabolic processes allows organisms to adapt to changes. Phase separation or MLOs formation is extremely sensitive to physicochemical cues. Even tiny changes in the local abundance of MLOs core components, post‐translational modifications (PTMs), RNA modifications, energy input, or physicochemical conditions (pH, salt ionic strength, temperature), etc., exert a significant regulation on the properties of phase separation.

### 4.1 Intracellular Concentrations of Core Components and Physicochemical Conditions

Phase separation is a kind of density transition that occurs spontaneously when the core components reach a threshold concentration.^[^ ^114^ ^]^ Direct evidence comes from an in vitro phase‐separating system in which hnRNPA1 spontaneously forms droplets in a concentration‐dependent manner. hnRNPA1 accumulation via blockade of nuclear import also leads to spontaneous SG assembly in cells.^[^ ^27^ ^]^ During oocyte‐to‐embryo transitions, P granules dissolve in the perinuclear region and recondense in the cytoplasm. This process is regulated by a concentration gradient. Likewise, asymmetric segregation of P‐granules into germline founder cells is induced by the P granule component guanyl‐specific ribonuclease PGL1 concentration gradient.^[^ ^13^ ^]^ Additionally, the core component concentration has been implicated in regulating the assembly and size of other MLOs such as PML bodies, nuclear speckles, histone locus bodies, nucleoli, and Cajal bodies.^[^ ^18^ ^]^ Furthermore, the transcriptional control of RNA concentration contributes to the dynamic MLO properties observed in nuclear speckles, paraspeckles, nucleoli, and P granules.^[^ ^115^, ^116^, ^117^, ^118^ ^]^

Cellular physicochemical conditions, such as temperature, pH, and ionic strength can impose a more direct impact on phase separation.^[^ ^24^ ^]^ The reason could be that LLPS of charged IDRs is mediated by weak electrostatic interactions that are highly sensitive to pH and ionic strength.^[^ ^2^, ^24^ ^]^ Heat shock, for instance, can induce the aggregation of proteins that are not identical to SGs. However, these aggregates are dissolvable and reversible when the cells are no longer subjected to heat stress.^[^ ^15^, ^16^ ^]^ Small pH fluctuations can also alter LLPS properties. The translation termination factor SUP35 undergoes LLPS to form droplets and subsequent gel transition to sequester the termination factor at a lower pH. However, increasing the pH triggers gel dissolution and restarts translation.^[^ ^119^ ^]^

### 4.2 Posttranslational Protein Modifications

To date, over 200 PTM types have been identified that influence almost all aspects of normal cell biology and pathogenesis.^[^ ^120^ ^]^ Due to the lack of a stable structure, LCDs are more susceptible to diverse PTMs.^[^ ^121^, ^122^ ^]^ Among them, protein phosphorylation, methylation, acetylation, PARylation, and SUMOylation are common PTM types that have been extensively studied. PTMs add additional features to proteins, thereby changing the charge state, steric conformation, or bulkiness.^[^ ^120^, ^123^ ^]^ As a result, PTMs affect phase separation by strengthening or disrupting multivalent interactions between biomacromolecules undergoing phase separation and/or by including/excluding certain biomacromolecules into/from existing MLOs.^[^ ^123^ ^]^ Here, we provide some examples of how PTMs influence on phase separation.

Phosphorylation plays a crucial role in signal transduction cascades and modulates MLOs dynamics and structural integrity. Mechanistically, phosphorylation covalently bonds a negatively charged phosphoryl group to a serine/threonine/tyrosine hydroxyl group, which can promote or inhibit phase separation, depending on the protein context.^[^ ^19^, ^124^ ^]^ The phosphorylation level of Tau microtubule‐binding protein controls the prime time of phase separation and droplet size. No phase separation occurs without Tau phosphorylation.^[^ ^125^, ^126^ ^]^ Similar phase separation promotion effects were observed upon the phosphorylation of eukaryotic translation initiation factor 2*α* (eIF2*α*), fragile X mental retardation protein (FMRP),^[^ ^127^ ^]^ G3BP,^[^ ^128^ ^]^ heterochromatin protein 1*α* (HP1*α*),^[^ ^9^ ^]^ nephrin,^[^ ^37^ ^]^ and TIA‐1/TIAL RNA‐binding protein homolog (TIAR‐2).^[^ ^129^ ^]^ In contrast, the LCD of FUS contains several putative phosphorylation sites, and phosphorylation weakens the tendency of FUS towards phase separation and aggregation.^[^ ^130^ ^]^ Phosphorylation in either an LCD or a structured domain in TDP43 is sufficient to suppress phase separation.^[^ ^131^, ^132^ ^]^ Moreover, an interesting feature shows that kinases and phosphatases coexist in some MLO entities, suggesting that active phosphorylation–dephosphorylation cycles ensure MLOs structural integrity.^[^ ^133^, ^134^, ^135^ ^]^ For example, phosphorylation of maternal‐effect germline 3/4 (MEG3/4) by dual specificity tyrosine‐phosphorylation‐regulated kinase 3 (DYRK3) inhibits P granule assembly. However, dephosphorylation by protein phosphatase 2A (PP2A) promotes granule assembly.^[^ ^133^ ^]^ Furthermore, RNA polymerase II (RNAPII) is hyperphosphorylated by regulatory cyclin‐dependent kinases (CDKs) at an intrinsically disordered carboxy‐terminal domain, which favors RNAPII incorporation into splicing condensates but disfavors incorporation into transcription initiation condensates, implicating that phosphorylation can partition a protein into a particular condensate as a selection mechanism underlying condensate preference.^[^ ^136^ ^]^

Acetylation negates the positively charged *ε*‐amine of lysine through the addition of an acetyl group. Meanwhile, the hydrophobicity of the acetylated protein is increased.^[^ ^137^ ^]^ The DDX3 X‐linked (DDX3X) LCD can be acetylated at multiple lysine residues by cAMP‐response element‐binding protein (CREB)‐binding protein (CBP), which impairs DDX3X coacervation. However, more DDX3X can be recruited into SGs, thereby increasing SG volume after deacetylation by histone deacetylase 6 (HDAC6).^[^ ^138^ ^]^ Similarly, Tau phase separation is disrupted by lysine acetylation, which reduces its incorporation into SGs.^[^ ^139^, ^140^ ^]^ In addition, chromatin‐rich condensates are collapsible via dynamic acetylation of multiple lysine residues in histone proteins.^[^ ^141^ ^]^ However, the contrary result shows that acetylation in the RRMs of TDP43 abolishes its RNA interaction ability and drives TDP‐43 demixing into intranuclear liquid spherical shells of anisosome.^[^ ^34^ ^]^

Arginine residues are monomethylated or symmetrically/asymmetrically dimethylated by protein arginine methyltransferases (PRMTs), which transfer one or two methyl groups from S‐adenosylmethionine.^[^ ^142^ ^]^ Arginine methylation shifts the protein charge and hydrogen bonding. Meanwhile, methylation increases bulkiness and hydrophobicity.^[^ ^143^ ^]^ RBPs are enriched with RGG/RG motifs, in which arginine is preferentially methylated.^[^ ^144^ ^]^ Further, arginine methylation is directly linked to the suppression of phase separation by reducing cation (Arg)–*π* interactions, as exemplified in DDX4,^[^ ^39^ ^]^ FMRP,^[^ ^145^, ^146^ ^]^ FUS,^[^ ^147^ ^]^ G3BP1,^[^ ^148^ ^]^ hnRNPA2,^[^ ^74^ ^]^ and RAP55.^[^ ^149^, ^150^ ^]^ Oppositely, Histone methylation (H3K9me3) recruits abundant HP1*α* into heterochromatin condensates through multiple H3K9me3 reader chromodomains.^[^ ^151^ ^]^ Other evidences also indicate a promotional role for arginine methylation in ataxin 2,^[^ ^152^ ^]^ cold‐inducible RNA‐binding protein (CIRP),^[^ ^153^ ^]^ hnRNPA1,^[^ ^154^ ^]^ LSM4,^[^ ^155^ ^]^ and serpine1 mRNA‐binding protein 1 (SERBP1)^[^ ^156^ ^]^ phase separation.

Poly (adenosine 5’‐diphosphate‐ribose) (PAR) is a nucleic acid that covalently modifies proteins by PAR polymerases (PARP) upon DNA lesions.^[^ ^157^ ^]^ Similar to nucleic acids, PARylated proteins are negatively charged, and thus electrostatic forces mediate interactions with other positively charged LCDs, such as the RGG repeat‐containing FET family.^[^ ^158^, ^159^, ^160^ ^]^ Therefore, PARylation can seed phase separation de novo. PARylated proteins are commonly found in MLOs such as DNA repair foci, mitotic spindles, nucleoli, and SGs. PARylation levels affect the formation and dynamics of these MLOs.^[^ ^20^, ^49^, ^99^, ^161^ ^]^ For instance, hnRNPA1 PARylation at its LCD is necessary for nucleocytoplasmic translocation, whereas PAR binding promotes the association of hnRNPA1 with SGs through its PAR‐binding motif (PBM).^[^ ^161^ ^]^ PAR chains at DNA repair foci are essential for FUS recruitment and DNA damage condensate formation.^[^ ^28^, ^99^ ^]^

SUMOylation is an additional PTM that promotes phase separation. SUMOylation increases the size and number of sensory rhodopsin 2 (SOP2) condensates compared to the unmodified protein.^[^ ^62^ ^]^ PML proteins that nucleate PML bodies undergo massive SUMOylation and subsequently recruit client proteins into the PML core through SUMO‐SIM interactions.^[^ ^162^ ^]^ SUMOylation of telomere‐binding proteins condenses Alternative lengthening of telomeres (ALT)‐associated PML bodies (APBs), which further leads to telomere clustering.^[^ ^163^ ^]^

### 4.3 RNA and DNA Modifications

The existence of RNA modifications is now widely acknowledged which can change the charge, structure, and rigidity of RNA, RNA–protein, and RNA–RNA interactions.^[^ ^164^ ^]^ N^6^‐methyladenosine (m^6^A) is a prevalent RNA modification that can be recognized by the YT521‐B homology (YTH) domain of YTH m^6^A RNA binding protein 1/2/3 (YTHDF1/2/3) proteins. Previous studies showed that mRNA with multiple m^6^A modifications can dramatically enhance YTHDF1/2/3 phase separation. Then, m^6^A‐mRNA–YTHDF complexes are partitioned into different endogenous MLOs, such as P‐bodies, SGs or neuronal RNA granules. The degree of mRNAs enrichment in SGs is directly in proportion to the number of m^6^A modifications per transcript even when controlling for transcript length. In addition, some mRNAs without m^6^A modification are also enriched in SGs, suggesting that mRNAs can also be recruited through m^6^A‐independent mechanisms.^[^ ^165^, ^166^ ^]^ An argue study indicates that mRNA length, rather than m^6^A modifications, has a promotion effect on mRNA partitioning in SGs.^[^ ^167^ ^]^ Additionally, m^6^A modifications were shown to repress RNA binding to G3BP1/2, ubiquitin‐specific protease 10 (USP10), Caprin‐1, and RNA‐binding motif 4 (RBM4) which are linked to the formation of SGs.^[^ ^168^ ^]^ Thus, the inconsistent observations above implicate that the role of m6A in SGs formation is still a debatable issue. DNA methylation (5‐methylcytosine) at CpG islands enhances heterochromatin formation, which is mediated by methyl‐CpG‐binding protein 2 (MeCP2) phase separation.^[^ ^169^ ^]^

### 4.4 Nonequilibrium Tuning by Energy Input

LLPS‐driven formation of biomolecular condensates is a thermodynamic equilibrium process. The active components flux into or out of condensates is halted when an equilibrium state is reached, despite the passive exchange of components is still ongoing due to the weak, short‐lived interactions.^[^ ^170^, ^171^ ^]^ However, life activities are highly dynamic, which requires a nonequilibrium state to maintain biomolecular condensates in active liquid phases for performing physiochemical reactions. Cells have adopted multiple energy‐consuming strategies fueled by enzymatic processes to keep phase‐separated condensates away from static equilibrium.^[^ ^170^, ^171^ ^]^ For one thing, this can potentially restrain condensates from deleterious solidification or aggregation which has been seen in age‐dependent diseases, such as irreversible *α*‐Synuclein aggregates in Parkinson's disease,^[^ ^172^ ^]^ or FUS^[^ ^28^ ^]^ and TDP‐43^[^ ^80^ ^]^ aggregates in ALS.

It was suggested that ATP supply is necessary for maintaining liquid‐like behavior in nucleoli and SGs.^[^ ^25^, ^29^, ^128^, ^173^ ^]^ Dramatically decreased ATP levels can induce a liquid‐to‐solid transition and change the overall cytoplasmic state in bacteria and yeast.^[^ ^174^, ^175^ ^]^ ATP‐related proteins such as helicases and chaperone proteins are commonly implicated in controlling the material properties of RNP granules.^[^ ^29^, ^34^, ^176^, ^177^, ^178^ ^]^ A conserved RNA helicase, DDX6, prevents the polymerization of RNP granules into nondynamic solids.^[^ ^176^, ^177^ ^]^ Vasa‐type RNA helicases preserve germ granule integrity.^[^ ^25^ ^]^ Heat shock protein 90 (HSP90) family chaperones have ATP‐dependent activity and are implicated in maintaining the liquidity of TDP43 phase separation and preventing TDP43 aggregation.^[^ ^34^ ^]^ These observations indicate that the maintenance of dynamic MLOs properties is ATP driven. However, the specific ATP‐driven processes involved in MLOs formation are currently unclear.

## 5 LLPS in Cancer

Cancer development and progression is a dynamic process involving several gene‐environment interactions. As well‐documented elsewhere, cancer cells exhibit several key hallmarks, including sustained proliferative signaling, growth suppressor evasion, cell death resistance, replicative immortality, angiogenesis, invasion, and metastasis.^[^ ^179^, ^180^ ^]^ Aberrant LLPS levels have been used to interpret the mechanisms underlying these cancer phenotypes. The outstanding advances are listed in **Table** **1** and are summarized below.

**Table 1:** Dysregulated condensates in cancer

Condensates	Biomolecules	Biological role	Cancer types	Refs.
Signaling puncta	EML4‐ALK fusion	RAS signaling overactivation	Non‐small‐cell lung cancer, ovarian cancer	[188, 189]
	CCDC6‐RET fusion	RAS signaling overactivation	Non‐small‐cell lung cancer, papillary thyroid cancer	[188, 189]
	SHP2 mutants	RAS signaling hyperactivation	Esophagus cancer	[190]
	DnaJB1‐PKAcat fusion	Tumorigenic cAMP signaling	Atypical liver cancer fibrolamellar carcinoma	[191]
	DACT1	WNT signaling inhibition	Breast and prostate cancer	[195]
Glycogen compartments	G6PC	YAP signaling activation	Liver cancer	[194]
Stress granules	YB1	Cell proliferation	Sarcoma	[196]
	KRAS mutants	Chemoresistance	Pancreatic cancer	[198]
	DDX3X	Tumorigenesis	Medulloblastoma	[276]
	mTORC1	Cancer cell survival	Multiple cancer types	[135, 200]
	Astrin	Protect cancer cells from apoptosis	Breast and lung cancer	[199]
Amyloid bodies	rIGSRNA	Tumor growth repression	Breast, lung, and prostate cancer	[202]
Perinucleolar compartments	PNCTR	Malignant transformation of cancer cells	Multiple cancer types	[204]
Nuclear speckles	SPOP mutants	Defect in oncoproteins degradation	Prostate cancer	[206]
Nuclear paraspeckles	NEAT1	Chemoresistance	Multiple cancer types	[207]
Amyloid fibrils	p53 mutants	Oncogenicity transformation of p53	Multiple cancer types	[208, 209, 210]
Heterochromatin	HP1*α*	Telomere elongation	Breast cancer	[9, 213]
Cajal bodies	Telomeric RNA	Telomere elongation	Multiple cancer types	[214, 215, 216, 217, 218]
PML bodies	TRF2, RAD52	Telomere elongation	Sarcoma	[219, 220, 221]
DNA repair foci	PARP1, FUS, TAF15, EWS	DNA damage repair	Multiple cancer types	[28, 223]
	MRN complex, *γ*H2AX	DNA damage repair	Multiple cancer types	[225, 226]
	MRN complex, DilncRNAs	DNA damage repair	Multiple cancer types	[226]
	53BP1	DNA damage repair	Multiple cancer types	[227]
NP bodies	NORAD, PUM1/2	DNA damage repair	Colon cancer	[230]
Transcription condensates	CDK7, CDK12, CDK13	Oncogenic transcription	Multiple cancer types	[17, 274, 275]
	YAP, TAZ	Oncogenic transcription	Breast cancer	[236, 237]
	ENL mutants	Oncogenic transcription	Wilms tumor	[238]
	EWS‐FLI1 fusion	Oncogenic transcription	Ewing sarcoma	[245]
	AKAP95	Oncogenic transcription and splicing	Multiple cancer types	[239]
Super‐enhancers	TAL1	Leukemogenic genes transcription	T cell leukemia	[240]
	BRD4, MED1	Oncogenic transcription	Multiple myeloma, liposarcoma	[241, 248]
	HOXB8	Oncogenic transcription	Osteosarcoma	[242]
	YTHDC1, m6A‐mRNAs	Gene transcription and mRNA processing	Acute myeloid leukemia	[243]
	NUP98‐HOXA9 fusion	Leukemogenic genes transcription	Leukemia	[246]
	FUS‐CHOP	Oncogenic transcription	Myxoid liposarcoma	[248]

### 5.1 Sensing Misregulated Signaling and Transduction

**Figure 4:**
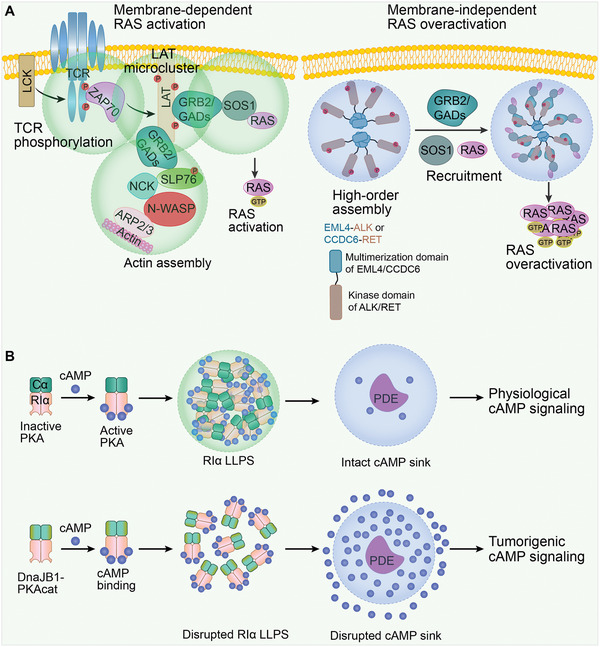
Misregulated signaling and transduction by LLPS in cancer. A) Left, membrane‐dependent RAS activation. A series of LLPS events results in RAS activation. Phosphorylated T cell receptor (TCR) activates the membrane kinase ZAP70, which then phosphorylates the tyrosine residues on linker for the activation of T cells (LAT). In turn, LAT microclusters form through enriching growth factor receptor‐bound protein 2 (GRB2) and GRB2‐related adaptor downstream of Shc (GADs). Subsequently, SOS1 is recruited for RAS activation. In addition, the actin effectors, non‐catalytic region of tyrosine kinase (NCK), neural Wiskott‐Aldrich syndrome protein (N‑WASP), and actin related protein (ARP) 2/3 complex can also be recruited for actin filament assembly. Right. membrane‐independent RAS overactivation. The oncogenic fusion protein, EML4‐ALK or CCDC6‐RET, obtains multimerization domains of echinoderm microtubule‐associated protein‐like 4 (EML4) or coiled‐coil domain‐containing protein 6 (CCDC6). These proteins lose membrane‐targeting sequences of anaplastic lymphoma kinase (ALK) or rearranged during transfection (RET) to form de novo membraneless cytoplasmic condensates. The RAS‐activating complexes GRB2/SOS1 are then concentrated in condensates, which leads to RAS overactivation. B) Upper, physiological cAMP signaling. In normal cells, RI*α*, a regulatory subunit of 3’, 5’‐cyclic adenosine monophosphate (cAMP)‐dependent protein kinase A (PKA), is capable of forming condensates and acting as a dynamic cAMP buffer. PKA activity is retained in the PDE sink. Lower, tumorigenic cAMP signaling. In fibrolamellar carcinoma, the native N‐terminus of PKA‐C*α* is replaced by the J‐domain of DnaJB1. The resulting DnaJB1‐PKAcat fusion oncoprotein interferes with RI*α* LLPS, thus disrupting cAMP compartmentation by PDE. As a result, cAMP signaling is highly active.

Cellular behavior and homeostasis are governed by diverse signaling pathways. Dysregulation of these pathways contributes to malignant cancer cell behavior.^[^ ^179^ ^]^ Early observations showed that membrane receptors and their corresponding signaling molecules can assemble into 2D clusters during signaling.^[^ ^181^, ^182^ ^]^ Mounting evidence suggests that LLPS could explain the high‐order assembly of these receptors. A well‐studied example is the formation of transmembrane clusters during T‐cell receptor (TCR) signaling transduction in immune cells.^[^ ^55^, ^183^, ^184^ ^]^ When TCRs are activated, the zeta chain of TCR‐associated protein kinase 70 (ZAP70) phosphorylates tyrosine residues in LAT. Then, multivalent SH‐containing protein family members, including GRB2, GRB2‐related adaptor downstream of Shc (GADs), and phospholipase C (PLC)‐*γ* are attracted to form membranous condensates.^[^ ^185^ ^]^ Subsequently, SOS1 is recruited for RAS signaling activation.^[^ ^55^, ^184^ ^]^ Alternatively, the SH2 domain‐containing leukocyte protein of 76 kDa (SLP76) binds to GRB2 or GADs to initiate the recruitment of Nck and N‐WASP actin effectors and the actin‐related protein 2/3 (ARP2/3) complex for actin filament assembly.^[^ ^184^, ^186^, ^187^ ^]^ Thus, LLPS seems to kinetically initiate higher‐order RAS signaling by preventing spontaneous SOS1 membrane localization from RAS activation (**Figure** **4**A).

However, membrane LLPS‐controlled RAS signaling is disrupted by chimeric receptor tyrosine kinase (RTKs) oncoproteins. The oncogenic RTKs, echinoderm microtubule‐associated protein‐like 4 (EML4)‐anaplastic lymphoma kinase (ALK), and coiled‐coil domain‐containing protein 6 (CCDC6)‐rearranged during transfection (RET) acquire multimerization domains of EML4/CCDC6 and lose the ALK/RET membrane‐targeting sequence, forms de novo membraneless cytoplasmic granules. These granules can serve as a subcellular platform to concentrate the RAS‐activating complex GRB2/SOS1. Thus, RAS signaling is activated in a lipid membrane‐independent manner (Figure 4A).^[^ ^188^, ^189^ ^]^ SH2 domain‐containing protein tyrosine phosphatase 2 (SHP2) is a major scaffold protein for RAS activation. Both increased and decreased phosphatase activity in SHP2 mutants can increase cancer risk. The explanation to this illusive manifestation underlies the gained LLPS of SHP2. SHP2 mutants are endowed with an open conformation in its PTP domain for LLPS capability. Meanwhile, additional wild‐type SHP2 is recruited and activated in LLPS to hyperactivate RAS signaling.^[^ ^190^ ^]^

LLPS also allows key signaling pathway components to concentrate rapidly in the cytoplasm, as demonstrated by the transduction of 3’,5’‐cyclic adenosine monophosphate (cAMP)‐dependent protein kinase A (PKA) signaling.^[^ ^191^ ^]^ PKA exists as a tetrameric holoenzyme consisting of a regulatory (R) subunit dimer and a pair of catalytic (C) subunits. The R subunit is a cAMP receptor. Of the four non‐redundant R subunits, RI*α* is ubiquitously expressed and is necessary for proper PKA activity. Aberrant RI*α* expression is correlated with tumorigenesis and tumor growth.^[^ ^192^ ^]^ RI*α* can undergo LLPS to form biomolecular condensates that spatially sequester high cAMP levels and retain high PKA activity in cells.^[^ ^191^ ^]^ In fact, discretely positioned phosphodiesterase (PDE) can function as a sink to compartmentalize cAMP in many cellular locations, thereby spatially and temporally regulating cAMP dynamics.^[^ ^193^ ^]^ In atypical liver cancer fibrolamellar carcinoma, the oncoprotein fusion between DnaJ Hsp40 member B1 (DnaJB1) and the PKA catalytic subunit (PKAcat), DnaJB1‐PKAcat, which explicitly abolishes RI*α* LLPS and leads to increased cAMP levels in PDE sinks and tumorigenic cAMP signaling (Figure 4B).^[^ ^191^ ^]^

Aberrant LLPS has also been implicated in other misregulated signal transduction pathways. Glucose 6‐phosphatase (G6PC), a regulator of hepatic glycogenolysis, is frequently downregulated in premalignant liver lesions, leading to glycogen accumulation. Condensed glycogen compartments are spontaneously formed from accumulated glycogen by LLPS. Further observations indicate that Laforin‐macrophage stimulating 1/2 (MST1/2) complexes assemble in glycogen liquid droplets to relieve MST1/2 inhibition on yes‐associated protein (YAP), thereby promoting malignant cell transformation.^[^ ^194^ ^]^ Transforming growth factor *β* (TGF‐*β*) functions as a main agitator of cancer invasion and metastasis. The TGF‐*β* target gene, the disheveled binding antagonist of *β*‐catenin 1 (DACT1), forms biomolecular condensates in the cytoplasm to sequester casein kinase 2 (CK2) and repress WNT signaling. LLPS of DACT1 is critical for breast and prostate cancer cell metastasis to bones, where DACT1 condensates exist.^[^ ^195^ ^]^

### 5.2 Cancer Cell Fitness Advantage

Hypoxia, high levels of reactive oxygen species, high osmotic stress conditions, acidosis, and nutrient starvation are typical microenvironment features in nearly all solid tumors due to the high metabolic demands of tumor hyperproliferation, which limits the available oxygen and blood supply.^[^ ^179^ ^]^ Previous studies indicate that adverse conditions can induce a cellular stress response and trigger SGs assembly to protect cancer cells from various stressors. SGs are formed by phase separation of core proteins, mainly G3BP1.^[^ ^27^ ^]^ Y‐box binding protein 1 (YB1) is highly expressed in human sarcomas and is correlated with cancer progression.^[^ ^196^ ^]^ YB1 was shown to directly upregulate G3BP1 translation and promote SGs formation.^[^ ^197^ ^]^ In addition, by stimulating the secretion of the lipid signaling molecule, 15‐deoxy‐Δ12,14 prostaglandin J2, KRAS mutations in pancreatic cancer cells enable paracrine SG elevation and confer resistance to stress stimuli and chemotherapeutic agents.^[^ ^198^ ^]^ ATPase activity in DDXs is essential for regulating RNA partitioning across different condensate types.^[^ ^178^ ^]^ DDX3X, a member of the RNA‐dependent DDX family, is implicated in RNA translation initiation and SG assembly. In medulloblastoma, DDX3X mutations impair RNA‐stimulated ATP hydrolysis, which leads to SGs hyperassembly. Consequently, SGs assembly itself contributes to broad translation inhibition. Thus, aberrant SGs assembly can be considered a tumorigenesis stimulator.^[^ ^178^ ^]^

The activity of Mammalian target of rapamycin complex 1 (mTORC1) is required for cancer cell survival, whereas chronic mTORC1 hyperactivation can sensitize cells to apoptosis. Thus, mTORC1 activity must be counterbalanced.^[^ ^199^ ^]^ Under oxidative or heat stress, mTORC1 is sequestered in SGs. mTORC1 reactivation is directed by SGs disassembly.^[^ ^135^, ^200^ ^]^ DYRK3 is a regulator of SGs formation. When stress is persistent, DYRK3 remains inactive and condenses mTORC1 in SGs. Once the stress is relieved, DYRK3 is activated by the chaperone protein HSP90, which allows SG dissolution to release mTORC1 for signaling by directly phosphorylating the mTORC1 inhibitor proline‐rich AKT substrate of 40 kDa (PRAS40).^[^ ^135^, ^201^ ^]^ Moreover, astrin, which is highly expressed in breast and lung cancer, confines the mTORC1 component raptor to SGs and restricts mTORC1 association during oxidative stimuli. mTORC1 activity is thereby inhibited, which rescues cancer cells from oxidative stress‐induced apoptosis.^[^ ^199^ ^]^

In addition, acidosis stress‐induced nuclear amyloid bodies can recruit proteins related to cell cycle progression and DNA synthesis. As such, cell proliferation and DNA synthesis are arrested, inducing cellular dormancy while remaining viable in response to an acidotic tumor microenvironment.^[^ ^202^ ^]^ Low‐complexity ribosomal intergenic RNA (rIGSRNA) drives the formation of amyloid bodies.^[^ ^203^ ^]^ Further, tumor growth was recovered upon silencing rIGS28RNA.^[^ ^202^ ^]^ A lncRNA PNCTR is markedly upregulated in multiple cancer cells. PNCTR can recruit and sequester pyrimidine tract‐binding protein (PTBP1) in a kind of nuclear body, perinucleolar compartment, via its hundreds of PTBP1‐specific motifs. By this way, PNCTR antagonizes PTBP1 splicing activity and drive the malignant transformation of cells.^[^ ^204^ ^]^

### 5.3 Evading Growth Arrest

Evading growth suppression conducted by endogenous tumor suppressors is an alternative way to accelerate cell growth.^[^ ^179^ ^]^ The tumor suppressor SPOP is a substrate adaptor of cullin3‐RING ubiquitin ligase (CRL3). SPOP recruits several oncoproteins, such as steroid receptor coactivator 3 (SRC3), myelocytomatosis oncogene (MYC), death domain‐associated protein (DAXX), androgen receptor (AR), and GLI3 (GLI family zinc finger 3). etc., to CRL3 for ubiquitination and proteasomal degradation.^[^ ^205^ ^]^ Additionally, SPOP is localized in liquid nuclear speckles, which retain their ubiquitination activity. Higher‐order SPOP oligomerization and multivalent interactions between SPOP and its substrates cooperatively trigger SPOP LLPS.^[^ ^61^, ^206^ ^]^ Typical mutations in SPOP disrupt phase separation and DAXX ubiquitination in prostate cancer,^[^ ^206^ ^]^ implying that defects in SPOP LLPS result in oncoprotein accumulation.

p53 is the most frequently studied tumor suppressor. p53 induces nuclear paraspeckle assembly by directly upregulating the scaffold lncRNA nuclear‐enriched abundant transcript 1 (NEAT1) in response to various oncogenic stimuli.^[^ ^115^, ^116^ ^]^ The paraspeckles act as tumor suppressors downstream of p53 to prevent early‐stage neoplasia.^[^ ^115^ ^]^ During tumor progression, paraspeckles are involved in chemoresistance, wherein NEAT1 promotes ataxia telangiectasia and Rad3‐related (ATR) signaling activation in response to replication stress. Besides, paraspeckle formation establishes a negative feedback loop to attenuate p53 signaling.^[^ ^207^ ^]^ Further studies indicate that oncogenic p53 transformation is associated with fibril formation of p53 mutants in cancers.^[^ ^208^, ^209^, ^210^ ^]^ For instance, the p53 R175H mutant, which has the highest occurrence in cancer patients, accelerates its aggregation and fibril formation. This state transition largely attributes to functional antitumor inactivation and oncogenicity transformation of p53.^[^ ^209^ ^]^ Similarly, the p53 R248Q mutant, the most common mutant found in breast cancers and is ranked among the strongest predictors of poor outcomes in ovarian cancer, forms mesoscopic protein‐rich clusters in cancer cells. Consequently, p53 R248Q clusters facilitate amyloid fibril nucleation.^[^ ^210^ ^]^

### 5.4 Telomere Maintenance

Telomeres are nucleoprotein structures formed by repetitive sequences of noncoding DNA and bound proteins at the end of the eukaryotic chromosome. Telomere structure is critical for genome stability by preventing chromosome end repair and degradation. Indeed, telomeres in normal somatic cells are progressively shortened by semiconservative DNA replication after each division cycle, eventually resulting in cell senescence or apoptosis.^[^ ^179^ ^]^ Oncogenic transformation can take advantage of telomere maintenance mechanisms (TMM) to achieve immortality.^[^ ^211^, ^212^ ^]^ An increasing number of studies show that LLPS widely participates in TMM and counteracts the deleterious effects of telomere shortening caused by an accelerated cell cycle in cancer cells. LLPS of HP1*α* is essential for forming liquid‐like heterochromatin domains and establishing telomeric silent chromatin by rapidly compacting DNA strands into puncta.^[^ ^9^, ^213^ ^]^ Thus, telomeric silencing favors dynamic telomere elongation.

Of the two TMMs, one depends on telomerase activity that is present in gametes, stem cells, and tumor cells. Human telomerase consists of a telomerase reverse transcriptase (hTERT) that is capable of synthesizing telomeric repeats de novo that lengthens a shortened telomere using telomeric RNA (hTR) as a template.^[^ ^211^, ^212^ ^]^ hTR can localize and accumulate in Cajal bodies in cancer cells via its CAB box signal.^[^ ^214^, ^215^ ^]^ What is more, over 25% of telomeres with increased hTR colocalize with Cajal bodies,^[^ ^216^ ^]^ thereby facilitating hTERT recruitment and telomere elongation.^[^ ^217^ ^]^ Telomerase‐dependent telomere extension is attenuated by mutating CAB box.^[^ ^218^ ^]^ These studies support the importance of Cajal bodies in telomerase‐dependent telomere extension.

Another TMM is recombination‐based ALT, which is employed by telomerase‐deficient cancer cells and sarcomas. In the ALT mechanism, telomere elongation components, mainly the shelterin protein TATA‐binding protein (TBP)‐related factor 2 (TRF2) and several DNA repair proteins such as recombinase RAD52 and Bloom syndrome helicase, are partitioned into PML bodies on telomeres to form APBs.^[^ ^219^, ^220^ ^]^ Telomeres cluster in the APB and elongate via mitotic DNA synthesis.^[^ ^221^ ^]^ Most APB components are marked by SUMOylation, SIM domains, or both. Therefore, APB behaves as a liquid‐like condensate that clusters telomeres in an SUMO‐SIM‐dependent manner.^[^ ^163^ ^]^

### 5.5 DNA Damage and Phase Separation‐Driven DNA Repair Foci

**Figure 5:**
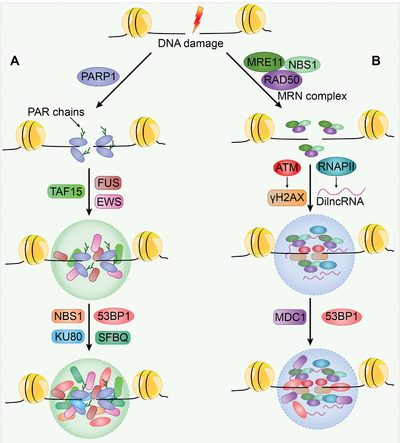
Model of DNA repair foci. A) Poly(adenosine 5’‐diphosphate‐ribose) polymerases (PARP)‐mediated DNA repair. Upon DNA break formation, PARylation is induced by PARP to recruit fused in sarcoma (FUS), Ewing's sarcoma oncoprotein (EWS), and TATA‐box binding protein associated factor 15 (TAF15), which drive DNA damage condensate assembly. DDR factors including ATP‐dependent DNA helicase II subunit 2 (KU80), Nijmegen breakage syndrome protein 1 (NBS1), p53 binding protein 1 (53BP1), and splicing factor proline and glutamine‐rich (SFPQ) are then attracted to the DNA repair foci. B) Meiotic recombination 11 (MRE11)‐RAD50 double strand break repair protein (RAD50)‐NBS1 (MRN) complexes formed repair foci. The MRN complexes recruit the apical protein kinase ATM for H2A histone family member X (H2AX) phosphorylation (*γ*H2AX). Meanwhile, RNA polymerase II (RNAP II) is also recruited to transcribe damage‐induced long noncoding RNAs (dilncRNAs). Consequently, these molecules drive the molecular crowding of DNA repair‐related proteins, including the mediator of DNA damage checkpoint protein 1 (MDC1) and 53BP1, for the assembly of DNA repair foci.

DNA damage events occur continuously in all organisms caused by several exogenous and endogenous stimuli. DNA damage can potentially have devastating effects on genomic instability, ultimately leading to cancer. Hence, a set of robust DNA damage repair (DDR) systems have evolved to deal with DNA damage.^[^ ^222^ ^]^ Recent studies demonstrated that transient and reversible condensates form at DNA repair foci to concentrate repair proteins and induce repair signaling. As exemplified in DNA damage sensor PARP1‐seeded liquid demixing, long PAR chains are synthesized by PARP1 at DNA damage sites. Then, FET proteins (FUS, TAF15, and EWS) are assembled to initiate DNA repair condensates.^[^ ^28^, ^223^ ^]^ FUS then attracts DDR factors including ATP‐dependent DNA helicase II subunit 2 (KU80), Nijmegen breakage syndrome protein 1 (NBS1), p53 binding protein 1 (53BP1), and splicing factor proline and glutamine‐rich (SFPQ) to the DDR foci (**Figure** **5**).^[^ ^224^ ^]^

Cells also utilize a different system for DDR via LLPS, i.e., meiotic recombination 11 (MRE11)‐RAD50 double‐strand break repair protein (RAD50)‐NBS1 (MRN) complexes formed repair foci. The apical protein kinase ataxia‐telangiectasia mutated (ATM) is recruited by MRN complexes to phosphorylate H2A histone family member X (H2AX), Then, the phosphorylated H2AX (*γ*H2AX) promotes the accumulation of several proteins, such as mediator of DNA damage checkpoint protein 1 (MDC1) and 53BP1 at DDR foci.^[^ ^225^ ^]^ Additionally, MRN complexes recruit RNAPII to transcribe damage‐induced lncRNAs (dilncRNAs). DilncRNAs then drive the molecular crowding of DDR‐related proteins, including 53BP1, into DDR foci that exhibit LLPS condensate properties.^[^ ^226^ ^]^ Uncoupled from upstream DNA damage detection and accumulation, 53BP1 confers LLPS behavior to downstream repair effectors such as p53 and the p53 coactivator USP28. Disruption of 53BP1 LLPS impairs p53 stability, p21 induction, and consequently, cell cycle arrest in response to DNA damage.^[^ ^227^ ^]^ Thus, 53BP1 phase separation connects two distinct condensates formed by DNA damage recognition factors and repair effector assembly (Figure 5).

In response to DNA damage, the levels of noncoding RNA activated by DNA damage (NORAD) increase for maintaining genomic stability through controlling the activity of RBPs.^[^ ^228^ ^]^ The RBP Pumilio proteins (PUM1 and 2) interact with and destabilize a large set of gene transcripts required for DNA repair, DNA replication, and mitosis.^[^ ^229^ ^]^ Owing to the abundance of PUMs binding sites, NORAD can nucleate the formation of PUM condensates (also termed NP bodies) to limit PUM activity by outcompeting thousands of other PUM‐binding transcripts in the human colon cancer. Therefore, NORAD‐driven PUMs phase separation provides a mechanism to maintain genomic stability.^[^ ^230^ ^]^ Additionally, NORAD also interacts with other DNA‐damage response component, RNA binding motif protein X‐linked (RBMX), through the strongest RBMX‐binding site. NORAD promotes RBMX‐mediated assembly of a RBP complex, term NORAD‐activated ribonucleoprotein complex 1 (NARC1), which contains the suppressors of genomic instability DNA topoisomerase I, Aly/REF export factor, and the pre‐mRNA processing factor 19 (PRPF19)‐cell division cycle 5 like (CDC5L) complex.^[^ ^231^ ^]^

### 5.6 Oncogenic Transcriptional Condensates

**Figure 6:**
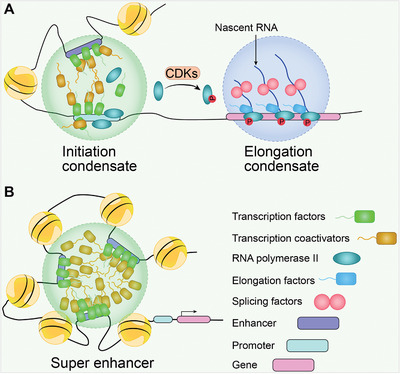
LLPS in gene transcription. A) Transcriptional condensates. Transcriptional condensates consist of initiation condensates and elongation condensates. Transcription factors (TFs) containing IDRs are prone to phase separation. TFs bind to regulatory elements such as enhancers and promoters to establish initiation condensates. Then, coactivators and unphosphorylated RNAPIIs are recruited. RNAPIIs are phosphorylated at the carboxy‐terminal domain by cyclin‐dependent kinases CDKs, which launches the transition from initiation condensates to elongation condensates. The elongation condensate is formed by phosphorylated RNAPIIs, elongation factors, nascent RNA, and splicing factors. B) Super enhancers (SEs). SEs are defined as clusters of enhancers enriched with TFs, coactivators, and RNAPIIs that drive robust genes expression in carcinogenesis.

Oncogenic dysregulation of transcription is a key driver of tumor initiation and progression. This process is called “transcriptional addiction.”^[^ ^232^ ^]^ Many proteins participate in transcription, including RNAPII, TFs, coactivators, and transcription elongation factors. Transcriptional condensates formed by LLPS offer a novel approach for understanding multimolecular assemblies.^[^ ^233^, ^234^, ^235^ ^]^ Live‐cell single‐molecule imaging revealed that TF LCDs condense into high‐concentration hubs at genomic loci. These condensates stabilize enhancer and promoter binding, recruit RNAPII, and efficiently initiate transcription. After proceeding to the elongation stage, differential phosphorylation by CDKs, especially CDK7 and CDK9, at the RNAPII carboxy‐terminal domain relocates RNAPII from the initiation condensates to elongation condensates that contain elongation factors, nascent RNA, and splicing factors (**Figure** **6**).^[^ ^136^ ^]^

LCDs in the activation domains of octamer‐binding transcription factor 4 (OCT4) and general control nonderepressible 4 (GCN4) promote condensate formation with the mediators MED1 and MED15 to activate gene expression.^[^ ^109^ ^]^ YAP and Tafazzin family protein (TAZ) activate TFs in various cancer types, form condensates and enhance the expression of YAP/TAZ‐specific proliferation genes through LLPS.^[^ ^236^, ^237^ ^]^ The TAZ condensates were shown as discrete nuclear puncta in breast cancer samples, but not in normal breast samples.^[^ ^237^ ^]^ Moreover, small in‐frame insertions or deletions in the YEATS domain of eleven‐nineteen lysine‐rich leukemia (ENL) enable ENL with greater self‐association and discrete, dynamic nuclear puncta formation in Wilms tumors. Such nuclear puncta functionally provide hubs for high concentrations of regulatory factors and cancer‐driven genes transcription.^[^ ^238^ ^]^ The transcription and splicing regulator, A kinase‐anchoring protein 95 (AKAP95), is frequently upregulated in multiple cancer types for promoting cancer cell growth and suppressing oncogene‐induced senescence. AKAP95 activity in splicing regulation and tumorigenesis is associated with condensate formation with proper liquidity and dynamicity in the nucleus.^[^ ^239^ ^]^

Oncogene activation is often involved in the phase‐separated formation of superenhancers (SEs). SEs are enhancer clusters that are occupied by numerous transcriptional enhancers and regulatory elements that drive robust gene expression in multiple cancers (Figure 6).^[^ ^17^ ^]^ In T cell leukemia, heterozygous somatic mutations of T‐cell acute lymphocytic leukemia protein 1 (TAL1) oncogene enhancer can introduce novel binding motifs for the TF myeloblastosis oncogene (MYB). By this way, a SE is created to recruit additional transcriptional apparatus, including the coactivator acetyltransferase CBP and core components of the leukemic transcriptional complex that contains GATA‐3, RUNX1, and TAL1. Consequently, TAL1 oncogene is activated to promote leukemogenic progression.^[^ ^240^ ^]^ Oncogenic SEs have been identified in multiple myeloma cells. In contrast to regular enhancers, bromodomain‐containing 4 (BRD4) and MED1 co‐occupy thousands of enhancers (i.e., SEs) associated with active oncogenes, including MYC.^[^ ^241^ ^]^ Homeobox B8 (HOXB8) and FOS‐like 1 (FOSL1), which are master TFs of core regulatory circuitry (CRC), mediate liquid‐like puncta formation of CRC in cell nuclei. Interfering with CRC phase separation via HOXB8 depletion or pharmacological inhibition restrains chromatin accessibility at SEs loci and disrupts RNAPII elongation of SE‐driven genes. As a result, osteosarcoma growth and metastasis are reduced.^[^ ^242^ ^]^ In addition, the m^6^A reader protein YTH domain containing 1 (YTHDC1) recognizes m^6^A‐mRNAs and forms nuclear condensates that are colocalized with nuclear speckles and SEs. These results imply a key function of YTHDC1 in regulating gene transcription and mRNA processing. An actual study demonstrated that YTHDC1 condensates protect oncogene mRNA from degradation. Compared to normal blood cells, YTHDC1 condensates are more abundant in acute myeloid leukemia (AML) cells to maintain cell survival and an undifferentiated myeloid state.^[^ ^243^ ^]^

### 5.7 LLPS of Fusion Oncoproteins Drives Oncogene Overactivation

**Figure 7:**
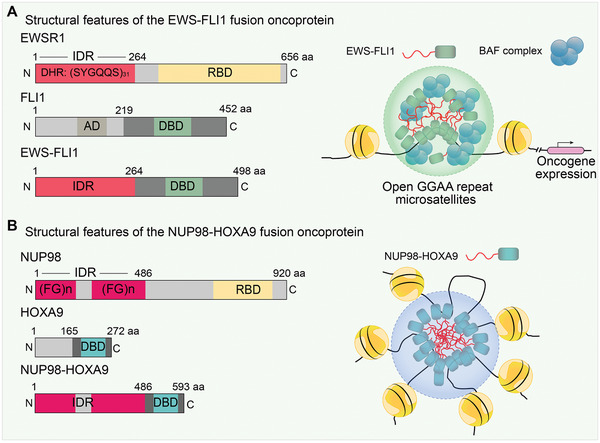
Gained LLPS of fusion oncoproteins drive oncogenes overactivation. The structural features of the two typical fusion oncoprotein EWS‐FLI1 and NUP98‐HOXA9 are shown on the left. Both oncoproteins are endowed with LLPS and transcription regulatory element binding ability. A) In Ewing sarcoma (EWS), EWS‐FLI1 establishes condensates at tumor‐specific GGAA repeat microsatellites and specifically recruit BRG1/BRM‐associated factor (BAF) complexes for oncogenes transcription. B) In leukemia, NUP98‐HOXA9 occupies chromatin and forms SEs‐like condensates which promotes leukemogenesis. EWSR1, EWS RNA binding protein 1; FLI1, friend leukemia virus integration site 1; NUP98, Nucleoporin 98 kDa; HOXA9, Homeobox A9; DHR, degenerate hexapeptide repeat; RBD, RNA binding domain; AD, activation domain; DBD, DNA‐binding domain.

Gene fusion results from merging distinct genes or gene fragments via structural chromosomal rearrangements. Many oncoproteins generated by gene fusion are strong drivers of tumorigenesis.^[^ ^244^ ^]^ Intriguingly, gain of LLPS ability has been implicated in the cancer pathology of such fusion oncoproteins.^[^ ^188^, ^245^ ^]^ In Ewing sarcoma, the well‐characterized oncoprotein EWS‐FLI1, which is produced by the fusion of the N‐terminal IDRs of EWS RNA binding protein 1 (EWSR1) and the DNA‐binding domain of friend leukemia virus integration site 1 (FLI1), acquires LLPS ability compared to wild‐type FLI1. BRG1/BRM‐associated factor (BAF) complexes are then specifically recruited by EWS‐FLI1 to tumor‐specific GGAA repeat microsatellites for target oncogenes transcription (**Figure** **7**A).^[^ ^245^ ^]^

Another remarkable example of fusion oncoproteins is NUP98‐HOXA9, which is recurrently detected in leukemia. This oncoprotein is the fusion of N‐terminal FG repeats in the nucleoporin 98 kDa (NUP98) and the chromatin targeting the homeodomain of homeobox A9 (HOXA9). NUP98‐HOXA9 is capable of establishing phase‐separated assemblies to enhance chromatin occupancy at leukemogenic genes and form SEs‐like binding patterns, thereby inducing leukemic transformation in mouse hematopoietic stem and progenitor cells. When phenylalanine in the chimeric FG repeats is substituted with serine, NUP98‐HOXA9 abolishes IDRs and loses LLPS and tumorigenic activity (Figure 7B).^[^ ^246^ ^]^ In line with this observation, other leukemogenic NUP98 fusions also recruit similar transcriptional machinery into biomolecular condensates.^[^ ^247^ ^]^ Furthermore, the oncogenic TF FUS‐CCAAT/enhancer binding protein homologous protein (CHOP) is localized in small nuclear puncta and colocalizes with the SE marker BRD4 in myxoid liposarcoma.^[^ ^248^ ^]^

## 6 Novel Therapeutics Underlying Phase Separation

**Figure 8:**
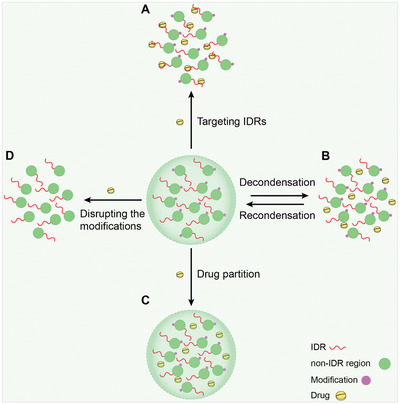
Proposed strategies to develop new cancer therapeutics by targeting biomolecular condensates. As illustrated in main text and listed in Table 2, the strategies include: A) Targeting intrinsically disordered regions (IDRs) that drive condensates formation; B) decondensation or recondensation dependent on the function of condensation, for example, by directly regulating the physiochemical properties of condensates; C) drug partitioning to concentrate drugs in condensates and increase treatment efficiency; and D) disrupting the modifications of condensate components, such as PTMs‐related enzymes or PTMs themselves, to affect condensates formation.

With the progression in understanding of LLPS biology in cancer, the feasibility of developing new cancer therapeutics has been proposed despite challenges exist. Rationally, two approaches have been adopted. One is to directly disrupt condensate formation by IDRs or physicochemical properties. The other is to selectively regulate LLPS proteins, such as PTMs (**Figure** **8** and **Table** **2**).^[^ ^249^, ^250^ ^]^

**Table 2:** Therapeutic targeting of condensates in cancer

Strategy	Reagent	Target	Effects	Refs
Targeting IDRs	Tin‐based metal cluster	TAF2 subunit of TFIID	Inhibit transcription initiation	[252]
	IIA4B20, IIA6B17, mycmycin‐1/2	c‐MYC	Inhibit MYC‐induced cell malignant transformation	[253, 254]
	YK‐4‐279	EWS‐FLI1 fusion	Block the interaction of EWS‐FLI1 and RNA helicase A and reduce EWS cells growth	[255]
	PRIMA‐1, APR‐246, ReACp53, Polyarginine	p53 mutants	Halt p53 mutants amyloid formation	[256, 257, 258]
	Elvitegravir	SRC1	Inhibit YAP oncogenic transcription	[259]
Decondensation or recondensation	1, 6‐hexanediol (HDO)	General solvent	Dissolve condensates directly	[79, 260]
	Lipoamide, Lipoic acid	General solvent	Dissolve FUS‐related SGs	[264]
	Bis‐ANS	Modulators of TDP‐43 condensates	Biphasic activity depends on the concentration	[265]
	C108	G3BP2	Reverse SGs‐mediated initiation of breast cancer	[262]
	Vinblastine	Microtubule	Dissolve SGs	[263]
	All‐trans retinoic acid, Arsenic trioxide	PML‐RARA fusion	Recover PML bodies in acute promyelocytic leukemia	[266]
	RNA silencing technology	RNAs	RNA‐driven formation of aberrant biomolecular condensates	[267]
Drug partition	Tamoxifen, Cisplatin, THZ1, JQ1	SEs	Actively partition into SEs and enhance the therapeutic activity	[261]
Disrupting the modifications	Olaparib	PARP1/2	Impairs PARylation‐related DNA repair condensates	[159]
	GSK‐626616	DYRK3	Inhibit PRAS40 phosphorylation and restrain mTORC1 signaling in SGs	[135, 269]
	SI‐2	NSD2	Abolish SRC3 methylation and sensitize bortezomib treatment	[271]
	JQ1	BRD4	Release the Mediator complex from SEs	[272]
	SGC0946	DOT1L	Inhibit histone H3K79 methylation and histone H4 acetylation	[273]
	THZ1	CDK7	Inhibit RNAPII phosphorylation	[17, 274]
	THZ531	CDK12 and CDK13	Inhibit RNAPII phosphorylation	[275]

### 6.1 Disrupting Condensate Formation

In traditional targeted therapies, the protein regions for intervention typically have clearly defined secondary and tertiary structures. However, the inherent thermodynamic instability of IDRs, which mediates condensate formation, has long been considered undruggable.^[^ ^251^ ^]^ Recent studies showed that small molecules bind to the IDRs of the oncogenic TFs MYC, c‐FOS, EWS‐FLI1, TAF2, and p53.^[^ ^251^, ^252^ ^]^ For example, a tin‐based metal cluster as identified as a selective inhibitor that binds to an IDR within the TAF2 subunit of TFIID.^[^ ^252^ ^]^ IIA4B20, IIA6B17, and mycmycin‐1/2 are highly specific for MYC and effectively inhibit MYC‐induced malignant cell transformation.^[^ ^253^, ^254^ ^]^ YK‐4‐279, which binds to a large IDR in EWS‐FLI1, blocks interactions between EWS‐FLI1 and RNA helicase A to reduce EWS cell growth.^[^ ^255^ ^]^

p53 mutants tend to form aggregates and thus lose tumor‐antagonizing ability. PRIMA‐1 and its structural analog, APR‐246, which are p53 reactivators, prevent mutant p53 aggregation and reverse the resulting amyloid state in cancer cells.^[^ ^256^ ^]^ In addition, the peptides ReACp53, polyarginine, and its analogs halt p53 mutant amyloid formation and rescue p53 inhibition in cancer cells.^[^ ^257^, ^258^ ^]^ The highly disordered steroid receptor coactivator 1 (SRC1) colocalizes with YAP‐transcriptional enhancer factor domain family member (TEAD) in phase‐separated transcriptional condensates. The anti‐HIV drug elvitegravir directly binds to SRC1 and effectively inhibits YAP oncogene transcription by disrupting SRC1 LLPS in SRC1/YAP/TEAD condensates.^[^ ^259^ ^]^

As for disrupting physicochemical properties, the aliphatic alcohol 1,6‐hexanediol (HDO) can dissolve many condensates by destroying weak hydrophobic protein‐protein or protein‐RNA interactions. Nevertheless, the non‐selectivity and cytotoxicity of HDO restrict its application.^[^ ^79^, ^260^ ^]^ Therefore, rather than general solvent, molecules that can be partitioned into cancer‐specific condensates to alter their properties are preferentially pursued. In a pioneering study, anti‐cancer compounds including the broad‐spectrum chemotherapeutic agent cisplatin, the topoisomerase inhibitor mitoxantrone, the estrogen antagonist tamoxifen, the CDK7 inhibitor THZ1, and the BRD4 inhibitor JQ1 were highly partitioned into SE‐associated biomolecular condensates, which enhance their activity.^[^ ^261^ ^]^ This type of partition is irrelevant of molecular targets, whereas *π*–*π* or *π*–cation interactions are physicochemical properties that favor molecule partitioning. Interestingly, cisplatin exerts anti‐cancer activity by dissolving SEs, indicating that changes in condensate properties may improve therapeutic outcomes.^[^ ^261^ ^]^

Another relevant research direction is to reverse the SG‐mediated initiation of breast cancer. C108, a lead compound from a high‐throughput drug screen, alleviates the function of the SGs core component G3BP2 in breast cancer initiation and enhances the therapeutic efficacy of chemotherapeutic agents.^[^ ^262^ ^]^ Microtubule integrity is essential for SGs assembly. The microtubule‐depolymerizing drug vinblastine can trigger SGs disappearance in cells.^[^ ^263^ ^]^ Additionally, the antioxidants lipoamide and lipoic acid promote the propensity of FUS‐related SGs to dissolve.^[^ ^264^ ^]^ Bis‐ANS and similar compounds are potent modulators of TDP43 condensates with biphasic activity. These compounds strongly promote LLPS at low concentrations due to their bivalence and highly hydrophobic naphthalene groups, whereas their negatively charged moieties disrupt liquid droplets via electrostatic repulsion‐driven decondensation at higher concentrations.^[^ ^265^ ^]^ The intrinsic features of these compounds provide some bases for designing more potent and selective compounds to modulate physicochemical properties of condensates. Reciprocally, determining the physicochemical principles of specific cellular condensates will lead to selective compound design.

An exceptional anticancer strategy is to promote condensate formation, i.e., recondensation. PML‐retinoic acid receptor *α* (RARA) fusion proteins have dominant‐negative effects on PML body assembly and cause transcriptional repression of differentiation genes in acute promyelocytic leukemia (APL). Restoring PML nuclear bodies by empirically discovered drugs, all‐trans‐retinoic acid, and/or arsenic trioxide is the mechanism underlying effective APL treatment.^[^ ^266^ ^]^

Furthermore, considering the importance of specific RNA‐driven formation of aberrant biomolecular condensates in cancer pathogenesis as mentioned above, it was proposed recently to develop novel cancer therapeutics, specifically in clinical settings, through targeting selected RNAs and thereby modulating phase separation using RNA silencing technology, such as antisense oligonucleotide, RNA interference (RNAi), clustered regularly interspaced short palindromic repeats (CRISPR)/Cas system.^[^ ^267^, ^268^ ^]^

### 6.2 Targeting the Modifications of Condensate Components

As aforementioned, PTMs in constituent proteins can affect LLPS dynamics, making this process an attractive intervention window. PARP1/2 inhibition by olaparib impairs PARylation‐related DNA repair condensates formation and DDR.^[^ ^159^ ^]^ DYRK3 kinase functions as a central dissolvase of multiple cellular condensates during mitosis. The DYRK3 inhibitor GSK‐626616 enables recondensation in cells.^[^ ^269^ ^]^ DYRK3 activity is also required for SGs dissolution to release mTORC1.^[^ ^135^ ^]^ Hence, DYRK3 inhibitors can restrain mTORC1 signaling. Parallel to DYRK3, CK2 represents another interesting target that is linked to SG disassembly via phosphorylation of the SGs‐nucleating protein G3BP1.^[^ ^270^ ^]^ Histone methyltransferase NSD2‐mediated LLPS of SRC3 delivers bortezomib resistance in multiple myeloma. Inhibitor SI‐2 abolishes SRC3 LLPS and sensitizes bortezomib treatment.^[^ ^271^ ^]^ These studies suggest that targeting disease‐related PTM enzymes that regulate LLPS is feasible and beneficial.

BRD4 and CDKs are core regulators of SEs and transcription condensates that serve as targets for repressing oncogene transcription.^[^ ^17^ ^]^ For example, BRD4 inhibition by JQ1 releases the mediator complex from a subset of SEs that drives leukemogenesis gene expression in AML.^[^ ^272^ ^]^ In mixed‐lineage leukemia (MLL), the disruptor of telomeric silencing 1‐like (DOT1L) can induce histone H3K79 methylation, which in turn facilitates histone H4 acetylation and BRD4 binding at SEs. Dual inhibition of DOT1L and BRD4 by SGC0946 and I‐BET, respectively, results in dramatic synergistic effects on MLL inhibition.^[^ ^273^ ^]^ THZ1 treatment inhibits CDK7‐mediated RNAPII phosphorylation and elongation factor recruitment, thus repressing oncogenes transcription, which maintains tumor cell identity and proliferation.^[^ ^17^, ^274^ ^]^ The CDK12 and CDK13 covalent inhibitor THZ531 reduces RNAPII hyperphosphorylation and transcription elongation. Concurrently, THZ531 substantially decreases DDR‐related gene expression and induces apoptosis in leukemia.^[^ ^275^ ^]^

The DDX family is critical for RNA metabolism from transcription to degradation in most living organisms. Most of these proteins are essential modulators of cellular protein‐RNA condensate formation, including SGs, P bodies, and P granules, *etc*. Abnormal DDXs expression is associated with cancer development, proposing them as attractive targets for cancer therapy.^[^ ^178^, ^276^ ^]^ In particular, the DDX3X inhibitors RK‐33 and NZ51, the eIF4A inhibitors silvestrol, hippuristanol, CR‐1‐31‐B, and pateamine A, and the phosphorylated DDX5 inhibitor RX‐5902 exhibit anti‐cancer activity.^[^ ^277^ ^]^ However, whether their anticancer activity is linked to condensates regulation needs to be further explored.

## 7 Concluding Remarks

Biomolecular condensates or MLOs formed by LLPS function as cellular machinery for organizing fundamental biological reactions and processes. In this review, we discussed the current status of biological LLPS, how it occurs, and how it is regulated. However, most of the current research on phase‐separated condensates in cells focuses on the descriptive phenomenon and biological functions, rather than the most basic physicochemical processes. We are thus far from fully understanding this complex field. Matching these physical and chemical properties with biological functions will be an important direction for future research. Furthermore, there are some questions remain elusive. What are the functional differences between LLPS‐formed assemblies and canonical protein complexes? What are the all constituents in each type of condensate and which of them are essential? Which factors do contribute to the dynamic condensation and decondensation? What are the physicochemical differences between the inner and outer condensate layers? Whether and how do different condensates communicate? How to interrogate the internal structures of condensates in living cells, tissues, and even organisms (**Table** **3**)?

**Table 3:** The current status of LLPS and the concepts for future study

Current status	The concepts and future directions
How it occurs: Multivalent interactions; Folded‐folded domains; IDR‐IDR domains; Folded‐IDR domains; Nucleic acids‐protein; Nucleic acids‐Nucleic acids.^[^ ^14^, ^37^, ^38^, ^43^, ^44^ ^]^ How it is regulated: Local abundance of MLOs core components; Post‐translational modifications (PTMs); RNA and DNA modifications; Non‐equilibrium tuning by energy input; Physicochemical conditions (pH, salt ionic strength, temperature).^[^ ^114^, ^123^, ^164^, ^298^ ^]^ Biological functions: Reaction crucible; Sequestration; Organizational hub; Specific transport.^[^ ^19^, ^114^, ^299^, ^300^ ^]^	The physicochemical properties of condensates and their connection to biological functions; The functional differences between LLPS‐formed assemblies and canonical protein complexes The all constituents in each type of condensate; Factors that contribute to the dynamic condensation and decondensation; The communication between different condensates; The internal structures of condensates in living cells, tissues, and even organisms.

Phase separation is a rather complex process that needs interdisciplinary collaboration to address these questions. There remain several limitations and challenges for studying biomolecular condensates in cells. Current approaches mainly depend on fluorescence microscopy to observe the mesoscopic properties (size, shape, viscoelasticity) of condensates. A major challenge is developing novel conceptual approaches, tools, and probes to induce, inhibit, or alter the physicochemical properties of specific condensates. Several optogenetic tools have been developed for controlling critical parameters and thus inducing intracellular phase separation spatiotemporally.^[^ ^63^, ^278^, ^279^, ^280^ ^]^ However, the conclusion to the experiment output should be drawn cautiously because of the exogenous introduction of tools. In addition, some microfluidic devices are employed for measuring nucleation kinetics by swiftly and precisely operating temperature, pressure, salt, pH, and shear force.^[^ ^281^, ^282^ ^]^ Accordingly, developing and applying microfluidic techniques will offer a new direction for exploring the LLPS kinetics and molecular driving forces. Chemical probes are widely used for studying dynamic processes within living cells. Similarly, rational design of condensates‐specific probes will be helpful for real‐time identifying and tracing LLPS state in living cells, or even in organisms with the assistance of single‐molecule ultrahigh resolution imaging. Moreover, in combination with the development of advanced high‐resolution imaging mass spectrometry, a quantitative understanding of biophysical/physiochemical properties of condensates will be achieved using probes.

Meanwhile, we also outlined the outcomes of aberrant phase separation in many cancers and potential therapeutics. Answering the above questions will improve our understanding of how physiological condensates convert to pathological condensates in cancer and other diseases. It is optimistic to envision that future in‐depth investigations into cancer‐associated phase separation and condensates will revolutionize our therapeutic applications in cancer.

## Conflict of Interest

The authors declare no conflict of interest.

## Author Contributions

J.R. and Z.Z. contributed equally to this work. J.R. conceived and drafted the manuscript. Z.Y.Z. and Z.Z. collected the information and summarized the Table. F.Z. and L.Z. provided valuable discussion and revised the manuscript. The authors read and approved the final manuscript.
